# Lupus nephritis in Africa: a systematic review of case reports and case series

**DOI:** 10.1186/s12882-025-04502-8

**Published:** 2025-10-07

**Authors:** Mohamed Jayte, Abdifitah Abdullahi Mohamed, Abdifatah Karshe, Awil Abdulkadir Abdi, Hanan Asad Hassan

**Affiliations:** 1 Department of Internal Medicine, Kampala International University, P.O. Box 7062, Kampala, Uganda; 2 Department of Microbiology, Kampala International University, Kampala, Uganda

**Keywords:** Lupus nephritis, Africa, Renal outcomes, Autoimmune disease, Developing countries

## Abstract

**Background:**

Lupus nephritis remains a severe manifestation of systemic lupus erythematosus across African populations, where diagnostic and therapeutic challenges significantly impact patient outcomes. This systematic review examines the clinical spectrum and management realities of this condition in African settings.

**Methods:**

Following PRISMA guidelines, we identified seven qualifying studies after screening 662 records from major databases. The analysis included five case reports and two case series documenting confirmed lupus nephritis cases across multiple African regions. We systematically evaluated patient characteristics, diagnostic approaches, treatment regimens, and clinical outcomes using standardized quality assessment tools.

**Results:**

Seven studies reporting 16 patients from four countries were included. The female-to-male ratio was 14:2 (7:1). Histological classifications included Class IV (5 cases), Class V (4 cases), Class VI (3 cases), and mixed IV/V (1 case), with one case diagnosed clinically. Two patients died and three progressed to end-stage renal disease requiring dialysis. Most cases occurred in young women, commonly presenting with nephrotic syndrome and hypertension. Proliferative and membranous patterns were the predominant biopsy findings. Corticosteroid-based regimens led to improvement in several patients, though outcomes varied regionally, with North African cases showing better responses than those from other regions where late presentation predicted poorer prognosis.

**Conclusion:**

These findings underscore both the clinical challenges and opportunities in managing lupus nephritis across Africa. The demonstrated variability in outcomes calls for coordinated efforts to establish context-appropriate diagnostic protocols and treatment guidelines, with particular attention to resource-limited settings.

**Clinical trials:**

Not applicable.

## Background

Lupus nephritis (LN) is a major cause of morbidity and mortality among patients with systemic lupus erythematosus (SLE), but it may also occur in isolation as biopsy-proven LN without full SLE classification criteria (21). Although SLE patients may develop renal impairment from diverse causes such as drug toxicity, infections, or hypertensive and diabetic nephropathy, LN remains the most important cause of CKD in this population (3). Studies consistently show that LN accounts for up to 60% of kidney involvement in SLE, contributes disproportionately to CKD progression, and is the leading driver of SLE-related mortality in the short-, mid-, and long-term after diagnosis (1,2,22).

The global picture of LN reveals important differences among populations. People of African, Hispanic, and Asian descent tend to develop more severe forms of the disease compared to white populations [1]. This is particularly concerning in Africa, where multiple challenges exist in managing LN effectively. Many patients face delayed diagnosis due to limited healthcare access, shortage of specialists, and high costs of diagnostic tests like kidney biopsies [2]. Additionally, treatment options are often limited by availability and affordability of medications.

In Africa specifically, the situation appears especially difficult. Research suggests that African SLE patients frequently develop kidney problems earlier in their disease course and experience faster progression to kidney failure [3, 4]. The reasons for this aggressive disease pattern likely involve a combination of genetic factors, environmental triggers, and healthcare system limitations. For example, certain gene variants common in African populations may increase susceptibility to severe LN [5]. At the same time, factors like frequent infections, limited access to routine care, and late referrals to kidney specialists contribute to worse outcomes.

Despite these challenges, the available data on LN in Africa remain scarce, mostly limited to case reports and small case series. A previous systematic review synthesized LN treatment and outcomes in Africa up to 2015 (9). Since then, new evidence has emerged. Following consensus guidance on when and how to update systematic reviews (23), we conducted this review to update knowledge, synthesize recent evidence, and critically appraise reported cases. Our specific aim was to describe the clinical features, treatment regimens, and outcomes of LN in Africa, highlighting disparities and unmet needs.

## Methodology

### Reporting and protocol registration

This systematic review was conducted according to PRISMA guidelines for systematic reviews of case reports and case series (5,6). The protocol was not prospectively registered, which is acknowledged as a limitation.

### Eligibility criteria

We included case reports and case series that described biopsy-proven or clinically diagnosed lupus nephritis (LN) in African patients with sufficient demographic, clinical, treatment, and outcome data. We excluded reviews, studies conducted outside Africa, duplicates, and reports without adequate clinical detail.

### Search sources and strategy

**Fig. 1:**
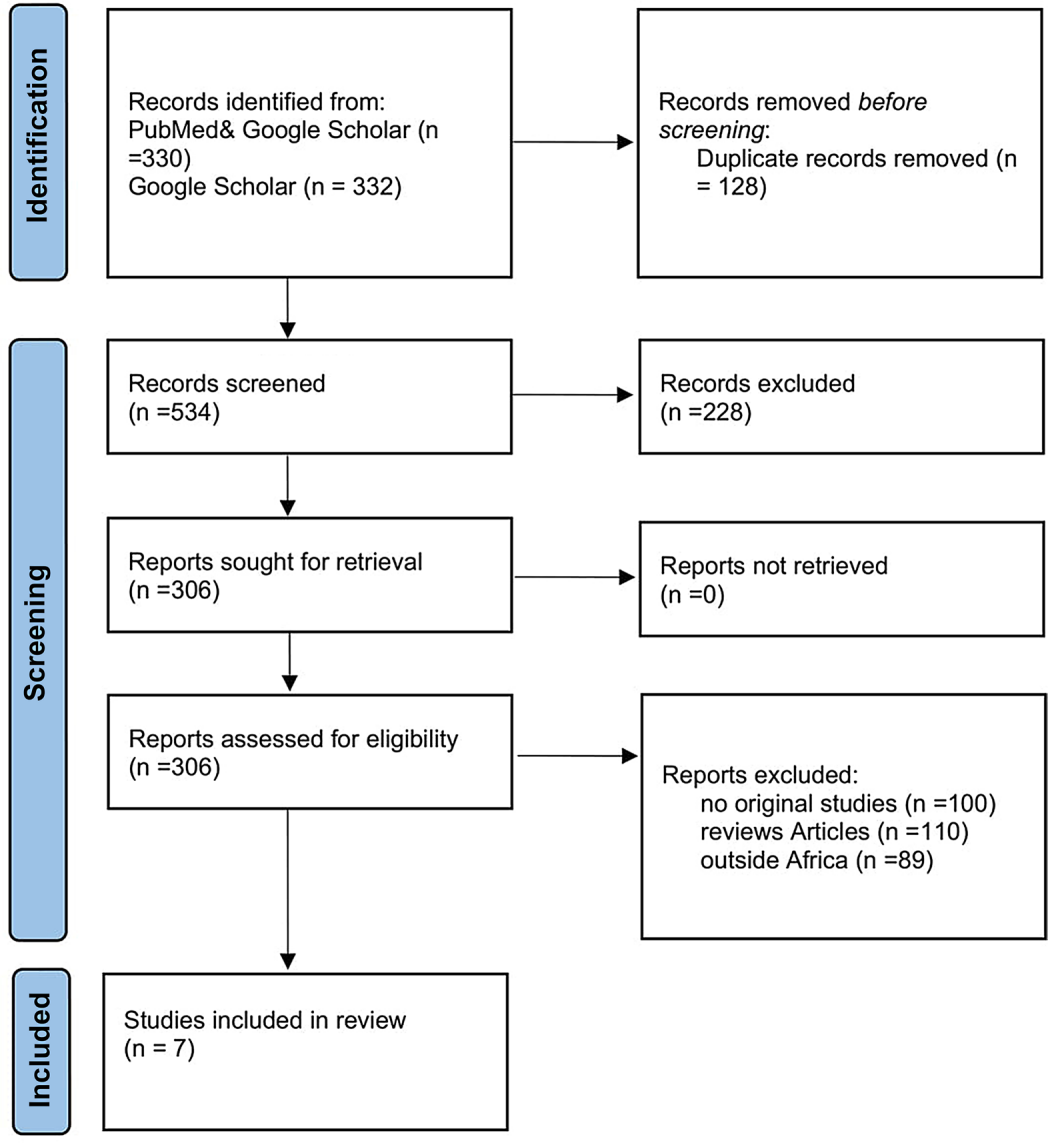
Showing PRISMA flow diagram showing the progression from 662 initial records to the 7 included studies

We systematically searched PubMed, African Journals Online (AJOL), and Google Scholar between January 2010 and April 2024. Search terms combined “systemic lupus erythematosus,” “lupus nephritis,” and “Africa,” adapted for each database. The search timeframe was chosen to build upon the previous systematic review of LN in Africa published in 2016 (9). We also screened the reference lists of eligible articles to identify additional reports. No language restrictions were applied during the initial search. However, case reports published in French and Arabic were excluded at full-text screening due to lack of translation resources, which may introduce bias as showing Fig. 1 Prisma chart.

### Selection

Two independent reviewers screened the titles/abstracts and full texts. Discrepancies in study selection were resolved by consensus.

### Data extraction

We developed a standardized data extraction form to collect information on patient demographics, clinical presentation, immunological findings, renal biopsy class, treatment regimens, and clinical outcomes.

### Risk of bias assessment

The methodological quality of included case reports was assessed using the CARE guidelines (7), while case series were appraised with the JBI critical appraisal checklist (8). All included studies met at least 70% of the relevant quality criteria. A detailed summary of study quality is provided in Supplementary Table 1.

### Data analysis

We performed a narrative synthesis of the included cases, supported by descriptive statistics where applicable. Meta-analysis was not attempted due to heterogeneity in study design, reporting, and outcomes.

### Ethical considerations

This review relied exclusively on previously published data; therefore, ethics approval was not required.

### Patient and public involvement

Patients or the public were not involved in the design, conduct, reporting, or dissemination of this research.

## Results

This systematic review identified and analyzed sixteen cases of lupus nephritis (LN) reported from four African countries: Tunisia, Nigeria, Tanzania, and Ghana. The cases included both individual case reports and case series published between 2018 and 2024. The patients were mostly female, with a female-to-male ratio of 7:1. Their ages ranged from 22 to 82 years, with clinical manifestations, laboratory findings, biopsy classification, treatment regimens, and clinical outcomes detailed in the respective studies in Table 2.

**Table 1:** Quality assessment summary of included studies

Study (Author, Year)	Checklist Used	Overall Score	Common Gaps
Hajji 2023 (Tunisia)	CARE	80%	No long-term follow-up
Mrabet 2024 (Tunisia)	CARE	78%	Missing outcome details
Sulaiman 2021 (Nigeria)	JBI	75%	Limited lab/immunological data
Ogala-Akogwu 2024 (Nigeria)	CARE	82%	No ethical approval reported
Ghosh 2023 (Tanzania)	JBI	76%	Short follow-up
Fredrick 2014 (Tanzania)	CARE	72%	Missing treatment details
Tannor 2018 (Ghana)	CARE	85%	Limited outcome data

**Table 2:** Clinical characteristics of lupus nephritis cases in Africa

Author, Year, Country	Age/Sex	Clinical Presentation	Biopsy Class (ISN/RPS)	Treatment	Outcome
Hajji 2023, Tunisia	38/F	Edema, fatigue, nephrotic proteinuria	V	Prednisone + HCQ	Remission (12 mo)
Mrabet 2024, Tunisia	82/M	Abdominal discomfort, HTN	Not done	Prednisone + HCQ	Remission (22 mo)
Sulaiman 2021, Nigeria	5 females (range 24–45y)	Nephrotic syndrome, HTN	IV (2 pts), V (2 pts), VI (1 pt)	Methylpred pulse + MMF	2 remission, 2 ESRD, 1 death
Ogala-Akogwu 2024, Nigeria	30/F	Oliguria, HTN, eGFR 11	VI	Steroids + MMF + dialysis	ESRD
Ghosh 2023, Tanzania	6 females	Edema, HTN, nephrotic syndrome	IV (2), V (3), III/V (1)	Steroids + MMF	Improved proteinuria
Fredrick 2014, Tanzania	28/F	Facial puffiness, nephrotic syndrome	III	Steroids	Partial remission
Tannor 2018, Ghana	22/M	Hematuria, oliguria, renal failure	IV/V	Prednisone + MMF + dialysis	Partial remission

Several case reports/series published in French and Arabic could not be included due to translation limitations, which may bias the results.

### Demographic characteristics

Out of the 16 patients, 14 (87.5%) were female, and 2 (12.5%) were male. The age distribution ranged from 22 years (a Ghanaian male) to 82 years (a Tunisian male), with most patients in their third to fifth decades of life. This aligns with the known epidemiological pattern of systemic lupus erythematosus (SLE) and LN, which predominantly affects women of reproductive age [6, 7].

### Clinical presentations

The predominant presenting symptoms included **nephrotic syndrome** (defined by significant proteinuria and edema), **hypertension**, and **generalized fatigue**. Patients from Nigeria and Tanzania, especially those in the case series by Sulaiman et al. [8] and Ghosh et al. [9], commonly presented with **recurrent leg swelling**, **facial puffiness**, **dyspnea**, and **hypertension**. The 38-year-old Tunisian woman presented with lower extremity edema, weight loss, and fatigue, symptoms consistent with nephrotic-range proteinuria and systemic illness [10]. Meanwhile, the 82-year-old Tunisian male reported vague systemic symptoms including abdominal discomfort and breathlessness [11].

One Ghanaian case by Tannor et al. involved a 22-year-old male who presented with **hematuria**, **oliguria**, and **signs of advanced renal failure**, including markedly reduced urine output and hypertensive urgency [12].

### Immunologic and laboratory findings

All patients tested **positive for antinuclear antibodies (ANA)**, confirming the diagnosis of systemic lupus erythematosus. ANA titers ranged from 1:160 in the Ghanaian case [12] to as high as 1:12,800 in the elderly Tunisian male [11]. **Anti-double-stranded DNA (anti-dsDNA)** antibodies were also reported in several patients, with titers up to > 800 IU/mL [11], supporting active lupus nephritis. Most cases exhibited **hypocomplementemia** (low C3 and C4 levels), which correlates with disease activity [10, 12, 13].

Renal function was compromised in multiple cases, particularly in the Nigerian and Tanzanian series, with **reduced eGFR** and elevated creatinine levels. For instance, the patient reported by Ogala-Akogwu et al. [14] had an eGFR of only 11.7 mL/min at diagnosis, signifying advanced renal damage.

### Lupus nephritis classification

Renal biopsies were performed in most cases and classified according to the **ISN/RPS lupus nephritis classification** system. The distribution was as follows:

- **Class IV (Diffuse Proliferative LN)**: Most common, reported in five cases, particularly among Nigerian and Tanzanian females [8, 9, 12].
- **Class V (Membranous LN)**: Observed in four patients, including the Tunisian case [10] and multiple Tanzanian patients [9].
- **Class VI (Advanced Sclerotic LN)**: Identified in three Nigerian patients, all of whom had poor renal outcomes, including two deaths [8, 13].
- **Class III (Focal Proliferative LN)**: Reported in one Tanzanian patient [10].
- **Mixed Class III/V**: Described in one Tanzanian patient [10].

Only one case, the 82-year-old Tunisian male, was diagnosed clinically without a renal biopsy. The Ghanaian case underwent biopsy, which demonstrated mixed Class IV and V lupus nephritis [11].

### Treatment regimens

The most commonly used treatment regimens included **high-dose corticosteroids** (either methylprednisolone or prednisolone), **mycophenolate mofetil (MMF)**, and **hydroxychloroquine (HCQ)**. In Tunisia, the patients received oral **prednisone** and **hydroxychloroquine**, with good clinical outcomes [10, 11]. Nigerian and Tanzanian patients were predominantly treated with **methylprednisolone pulses**, followed by oral steroids and **MMF** [8, 9]. The Ghanaian case also received **high-dose prednisolone** and **MMF** [12].

In advanced cases with kidney failure, **hemodialysis** was initiated, as seen in the Nigerian case described by Ogala-Akogwu et al. [13], who also continued immunosuppression despite renal impairment.

### Clinical outcomes

Clinical outcomes were highly variable and seemed to correlate with the LN class and renal function at diagnosis. In Tunisia, both patients achieved **clinical remission**, with no relapses after 12 and 22 months, respectively [10, 11]. In Nigeria, two patients with Class IV LN achieved remission, while three patients with Class V and VI LN experienced poor outcomes-two required **chronic hemodialysis**, and two died during the course of treatment [8, 13]. The Tanzanian series reported **significant improvement in proteinuria** in all six patients following immunosuppressive therapy, although long-term outcomes were not fully reported [9]. The Ghanaian patient achieved **partial remission**, with proteinuria reducing to 0.55 g/day after six months [12].

## Discussion

This review updates the 2016 systematic review of LN in Africa (9) by including newer cases published between 2016 and 2024. Our findings reinforce LN as a major cause of CKD and mortality in African SLE patients and provide additional evidence of regional disparities in presentation and outcomes.

Our analysis of lupus nephritis (LN) cases across Africa demonstrates patterns that both confirm and extend existing global understanding of this condition. The strong female predominance we observed aligns with established SLE epidemiology, though our cohort showed a slightly higher female-to-male ratio than the 6:1 reported in the multinational GLADEL cohort study of Latin American patients [12]. The frequent presentation with nephrotic syndrome corresponds with findings from the Euro-Lupus Nephritis Trial, where 58% of patients had nephrotic-range proteinuria at baseline [13].

Histopathological patterns in our African cases showed greater proportions of Class V disease than the 25% reported in the NIH LN registry [15], but similar Class IV prevalence to the 42% found in the ALMS trial [14]. These differences may reflect true population variation or later diagnosis, as suggested by the higher chronicity indices we observed compared to contemporary biopsy series from North America [14].

In the BLISS-LN trial (NEJM 2020), belimumab added to standard therapy improved renal outcomes, with 43% of patients achieving the primary efficacy renal response at week 104 compared with 32% in the placebo group, and 30% versus 20% achieving complete renal response [3], though direct comparison is limited by our retrospective design. The mortality and ESRD rates we documented exceed those from the recent systematic review by Tektonidou et al. [5], but align closely with data from the African Lupus Genetics Network [16].

The KDIGO 2024 guidelines provide updated recommendations for lupus nephritis management. For active Class III/IV (±V), initial therapy options include (i) mycophenolic acid analogues (MPAA) plus glucocorticoids, (ii) low-dose intravenous cyclophosphamide plus glucocorticoids, (iii) belimumab added to MPAA or low-dose cyclophosphamide, and (iv) calcineurin inhibitor-based combinations such as voclosporin. For maintenance, MPAA is the preferred agent, with treatment continued for at least 36 months. In Africa, however, most patients are limited to corticosteroids and MMF when available, with cyclophosphamide often used as a lower-cost alternative. Biologics such as belimumab and CNIs remain largely inaccessible, highlighting the gap between guideline recommendations and real-world practice.

Notable regional differences within Africa mirror findings from the multinational SLEGEI study, which identified significant outcome variations across different ethnic groups despite similar treatment approaches [16].

Health-system limitations also play a major role in the poor outcomes seen across African cohorts. The ISN Global Kidney Health Atlas (2023) documented severe gaps in kidney care delivery across Africa, including nephrologist density of less than 1 per million population (compared to a global average of 10), kidney biopsy services available in only ~ 30% of centers, intermittent availability of immunosuppressive drugs such as MMF and cyclophosphamide, and access to dialysis for fewer than 20% of patients in need. Most countries rely heavily on out-of-pocket funding, which further limits access to care as showing Table 3.

**Table 3:** Health-system indicators for kidney care in Africa (ISN-GKHA 2023)

Indicator	Africa average	Global average
Nephrologists per million	< 1	10
Kidney biopsy availability	~30%	> 80%
Immunosuppressant availability (MMF, CYC)	Intermittent	Standard
Dialysis access (patients who receive)	< 20%	> 70%
Funding model	Mostly out-of-pocket	Insurance/government

The particularly severe outcomes in West African cases may be partly explained by the high prevalence of APOL1 risk alleles, which have been associated with kidney disease susceptibility in these populations. However, given the small sample size and reliance on case-based reports, these findings should be interpreted as hypotheses requiring further study rather than definitive conclusions [16].

Another critical factor influencing outcomes is the limited access to newer therapies, such as belimumab and voclosporin, which have demonstrated improved renal responses in randomized trials. These agents are either unavailable or unaffordable in most African healthcare systems, leaving patients reliant on steroids and conventional immunosuppressants.

These findings underscore the conclusions of the 2019 EULAR recommendations for LN management, which emphasized the need for regionally adapted treatment guidelines [17]. They also support calls from the ISN Global Kidney Health Atlas for improved nephrology resources across Africa [12]. Future research should build on frameworks like the African Lupus Treatment Trial Group’s initiatives [15] to generate prospective, controlled data from these underserved populations.

### Limitations

This review has several limitations. First, the sample size is small and derived mainly from case reports and small case series, which may not be representative of the broader patient population. Second, there is heterogeneity in the reporting of clinical features, histology, and outcomes, making comparisons difficult. Third, publication bias likely favors the reporting of unusual or severe cases. Finally, long-term follow-up data were rarely available, limiting insights into chronic outcomes.

## Conclusion

In summary, this review highlights the severe and heterogeneous nature of LN in Africa, characterized by late presentation, frequent proliferative or membranous patterns, and poor outcomes in many regions. By updating the 2016 review (9), we provide evidence that regional disparities persist, with better outcomes in North Africa compared to West and sub-Saharan Africa. Addressing these gaps requires early detection programs, context-adapted guidelines, and stronger nephrology capacity across African healthcare systems.

## Data Availability

No datasets were generated or analysed during the current study.
